# Value of markers of systemic inflammation for the prediction of postoperative progression in patients with pancreatic neuroendocrine tumors

**DOI:** 10.3389/fendo.2024.1293842

**Published:** 2024-02-01

**Authors:** Liu Yang, Mengfei Fu, Li Yu, Hanyu Wang, Xiao Chen, Hui Sun

**Affiliations:** ^1^ Department of Endocrinology, Union Hospital, Tongji Medical College, Huazhong University of Science and Technology, Wuhan, China; ^2^ Hubei Provincial Clinical Research Center for Diabetes and Metabolic Disorders, Department of Endocrinology, Union Hospital, Wuhan, China; ^3^ Department of Emergency Medicine, Union Hospital, Tongji Medical College, Huazhong University of Science and Technology, Wuhan, China

**Keywords:** pancreatic neuroendocrine tumors, markers of systemic inflammation, Lymphocyte to monocyte ratio, biomarker, prognosis

## Abstract

**Background:**

Non-invasive prognostic predictors for rare pancreatic neuroendocrine tumors (PNETs) are lacking. We aimed to approach the prognostic value of preoperative systemic inflammatory markers in patients with PNETs.

**Methods:**

The clinical data of 174 patients with PNETs undergoing surgical treatment were retrospectively analyzed to explore the correlation of neutrophil to lymphocyte ratio (NLR), platelet to lymphocyte ratio (PLR), lymphocyte to monocyte ratio (LMR), and platelet to white blood cell ratio (PWR) with clinicopathological parameters and the progression of tumor after the operation. The optimal cutoff values for predictors and the area under the curve (AUC) of the receiver operating characteristic (ROC) were estimated. Univariate and multivariate Cox proportional hazards models were used to assess the relation between NLR, LMR, PLR, and progression-free survival (PFS), examined by the Kaplan–Meier and log-rank tests.

**Results:**

The scores of the NLR (*P* = 0.039) and PLR (*P* = 0.011) in the progression group were significantly higher than those in the progression-free group, and the LMR was significantly lower than those in the progression-free group (*P* = 0.001). The best cutoff values of NLR, LMR, and PLR before operation were 2.28, 4.36, and 120.91. The proportions of tumor progression in the high NLR group (*P* = 0.007) and high PLR group (*P* = 0.013) obviously increased, and the proportion of tumor development in the low LMR group was higher than that in the high LMR group (*P* < 0.001). The K-M survival curve showed that the progression-free survival rate was lower in the high NLR group (*P* = 0.004), the low LMR group (*P* < 0.001), and the high PLR group (*P* = 0.018). The results of the multivariate Cox proportional hazards model suggested that preoperative LMR (HR = 3.128, 95% CI: 1.107~8.836, *P* = 0.031) was an independent predictor of PFS.

**Conclusion:**

The markers of systemic inflammation, especially LMR, can predict the postoperative progression of PNETs.

## Introduction

Pancreatic neuroendocrine tumors (PNETs) are rare tumors of pancreatic endocrine origin and show an increasing rate of incidence, accounting for approximately 2% of pancreatic tumors (1, 2). Survival rates have not kept pace with incidence, and improved understanding, diagnosis, and treatments are urgently required. Metastasis, malignant transformation, and significant heterogeneity are hallmarks, and surgery is the main therapeutic approach (3, 4). Patients remain at risk of recurrence and metastasis post-surgery, and preoperative prediction of prognosis would aid treatment strategies. Disease grading and staging are valuable predictors of PNET survival (5) but are complex, invasive, and expensive. Therefore, there is an urgent need for convenient and effective prognostic markers.

Cancer progression is acknowledged to be influenced by a complex interplay of tumor inflammation, including low-level chronic inflammation, characterized by a sustained increase in inflammatory cells and proinflammatory mediators (6–8). Systemic inflammation may be evaluated by the neutrophil to lymphocyte ratio (NLR), platelet to lymphocyte ratio (PLR), lymphocyte to monocyte ratio (LMR), and platelet to white blood cell ratio (PWR), the assessments of which are simple, non-invasive, and low cost. Such ratios not only have predictive value in solid cancers but also are prognostic biomarkers for many malignancies, such as renal cell carcinoma, breast cancer, colorectal cancer, and pancreatic cancer (9–13). The above indexes are routinely measured; therefore, their clinical application value has gradually increased in recent years.

Due to the rarity of PNETs, there is a paucity of data exploring the relationship between systemic inflammatory factors and their prognosis. Several studies have confirmed NLR as a promising prognostic predictor for lymph node metastasis or recurrence in patients with PNETs. However, previous studies have mainly focused on NLR, and the effectiveness of other biomarkers, such as LMR, was not consistent (14–16). Therefore, it is necessary to further supplement clinical data for the prognostic value of markers of systemic inflammation in patients with PNETs after surgery.

This study intends to comprehensively explore the application value of NLR, PLR, LMR, and PWR in predicting the prognosis of PNETs so as to provide more abundant and comprehensive data support for clarifying the prognosis after tumor surgery.

## Patients and methods

### Study subjects

The data of PNET patients from the Union Hospital, Tongji Medical College, Huazhong University of Science and Technology from 2009 to 2021 were collected through the hospital’s electronic case system and retrospectively analyzed. One hundred eighty-two patients whose surgical specimens were pathologically examined and confirmed to be PNETs were included. The exclusion criteria were as follows: 1) presence of multiple endocrine neoplasia type 1 (MEN1), neurofibromatosis type 1 (NF1), or other genetic diseases; 2) hematological tests showed significant abnormalities such as too low or too high platelets, lymphocytes, etc.; 3) evidence of infection such as pyrexia, systemic inflammatory response, and other inflammatory conditions within 1 week before surgery; and 4) incomplete clinical data. Four patients were excluded due to incomplete data. One patient with primary hyperparathyroidism and pituitary tumors, one patient who had a blood routine that showed very low platelets, and two patients with severe preoperative inflection were excluded from the study. A total of 174 patients were ultimately included. This study was approved by the Committee for Medical Ethics of Union Hospital, Tongji Medical College, Huazhong University of Science and Technology.

### Follow-up

Patients were followed up through outpatient visits or by telephone until June 2022. Study endpoint events were tumor progression, including metastasis and recurrence, or death from any cause. Progression-free survival (PFS) was defined as the number of months from surgical treatment to tumor progression or to the date of final follow-up.

### Data collection

General patient data included age, sex, body mass index (BMI), smoking and drinking status, and history of past illness. Clinical data included routine blood examination and tumor markers such as carbohydrate antigen 125 (CA125), carbohydrate antigen 199 (CA199), and neuron-specific enolase (NSE) within 1 week prior to surgery, tumor size and function, location, pathological information, and functioning status. Tumors that overproduce hormones may be associated with distinct clinical syndromes and are referred to as functional; those that do not secrete hormones, secrete them in minimal quantities, or secrete peptides that do not result in an obvious syndrome (e.g., pancreatic polypeptide) are termed non-functional (3). The cutoff values for normal CA125, CA199, and NSE are less than 35 U/mL, 37 U/mL, and 16.3 μg/L, respectively.

### Statistical analysis

IBM SPSS Statistics software 26.0 and GraphPad Prism 7.0 were used for data analysis and plotting. For continuous variable data, such as age and BMI, the Kolmogorov–Smirnov test was used to analyze whether the data conformed to a normal distribution and expressed as mean ± standard deviation (*x* ± *S*). Comparisons were made by *t*-test. Non-normally distributed data such as inflammatory markers were expressed as median (M) and interquartile range (IQR) and compared by the Mann–Whitney *U* test. The chi-square test was utilized to evaluate count data, including gender, personal history, history of past illness, and tumor size. We used the receiver operating characteristic (ROC) curves, and the Youden index was calculated to find the optimal cutoff values for NLR, LMR, and PLR. The principle of the ROC curve is to assign multiple critical values to continuous variables, calculate the corresponding sensitivity and specificity at each critical value, and then plot a curve using sensitivity as the ordinate and 1-specificity as the abscissa. The best cutoff value refers to the best combination of sensitivity and specificity. The area under the curve (AUC) is defined as the area under the ROC curve. It is a measure of the model’s discriminatory power, where a higher AUC indicates better performance. The Kaplan–Meier curve and the log-rank test were used to analyze the relationship between NLR, LMR, and PLR with PFS rate. The Cox proportional hazards model was used to determine prognostic indicators by univariate analysis. Meaningful indicators were selected in the univariate analysis for multivariate analysis. A value of *P* <0.05 was considered to indicate statistical significance.

## Result

### General data and clinical pathological data

A total of 174 patients were eventually included in this study. The postoperative rates of tumor progression were 43/174 (24.71%), and 131/174 (75.29%) patients were progression-free. The mean patient age was 51.61, and 82 (47.13%) were men and 92 (52.87%) were women. The mean BMI was 23.91 kg/m^2^. Smokers and alcohol drinkers accounted for 10.92% and 8.05% of all patients, respectively. Eighteen patients (10.34%) had hypertension, 15 patients (8.62%) had diabetes, and 9 (6.43%) had a past history of other cancer types. Median NLR, PLR, LMR, and PWR were 1.76 (IQR, 1.39–2.35), 113.81 (IQR, 92–145.6), 5.19 (IQR, 3.82–6.79), and 37.45 (IQR, 29.83–45.84), respectively (**Table 1**).

**Table 1:** Baseline characteristics of patients with PNETs.

Parameters	Total (*n* = 174)
Age (years)	50.61 ± 12.91
Gender, *n* (%)	
Male	82 (47.13%)
Female	92 (52.87%)
BMI (kg/m^2^)	23.91 ± 3.66
Smoking, *n* (%)	
Yes	19 (10.92%)
No	155 (89.08%)
Drinking, *n* (%)	
Yes	14 (8.05%)
No	160 (91.95%)
Size, *n* (%)	165
>2 cm	76 (46.06%)
≤2 cm	89 (53.94%)
Location of the tumor, *n* (%)	168
Head, uncinate, and neck	78 (46.43%)
Body or tail	88 (52.38%)
Multiple	2 (1.19%)
Subtype, *n* (%)	
Functioning	78 (44.83%)
Non-functioning	96 (55.17%)
Histological grade, *n* (%)	158
G1	88 (55.70%)
G2+G3	70 (44.30%)
TNM staging, *n* (%)	171
I	64 (37.43%)
II	70 (40.94%)
III	5 (2.92%)
IV	32 (18.71%)
Progress, *n* (%)	
Yes	43 (24.71%)
No	131 (75.29%)
Inflammatory markers (median, IQR)	
NLR	1.76 (1.39, 2.35)
PLR	113.81 (92, 145.6)
LMR	5.19 (3.82, 6.79)
PWR	37.45 (29.83, 45.84)
Past history, *n* (%)	
Hypertension	18 (10.34%)
Diabetes	15 (8.62%)
Cancer	9 (6.43%)

PNETs, pancreatic neuroendocrine tumors; BMI, body mass index; NLR, neutrophil to lymphocyte ratio; LMR, lymphocyte to monocyte ratio; PLR, platelet to lymphocyte ratio; PWR, platelet to white blood cell ratio.

We analyzed the characteristics of the tumor, such as size, location, and pathological stage. The median tumor diameter was 2 cm (range: 0.2–13 cm). Tumor diameter was >2 cm in 76 cases (46.06%) and ≤2 cm in 89 cases (53.94%). More patients had non-functional than functional tumors (55.17% vs. 44.83%). Seventy-eight (46.43%) were located in the pancreatic head and neck, and 88 (52.38%) were located in the body and tail. Two (1.19%) patients had multiple tumors. Differentiation was graded histologically according to the World Health Organization (WHO) pathological grading standard for gastrointestinal and pancreatic neuroendocrine tumors (17). Eighty-eight cases were poorly differentiated (G1), 65 moderately differentiated (G2), and 5 well-differentiated (G3). Comprehensive clinical staging was performed according to the TNM staging criteria of the 8th edition of the American Joint Commission on Cancer (AJCC) for PNETs (18). Sixty-four patients (37.43%) had stage I, 70 (40.94%) stage II, 5 (2.92%) stage III, and 32 (18.71%) stage IV (**Table 1**).

### Markers of systemic inflammation and clinicopathological parameters

**Figure 1:**
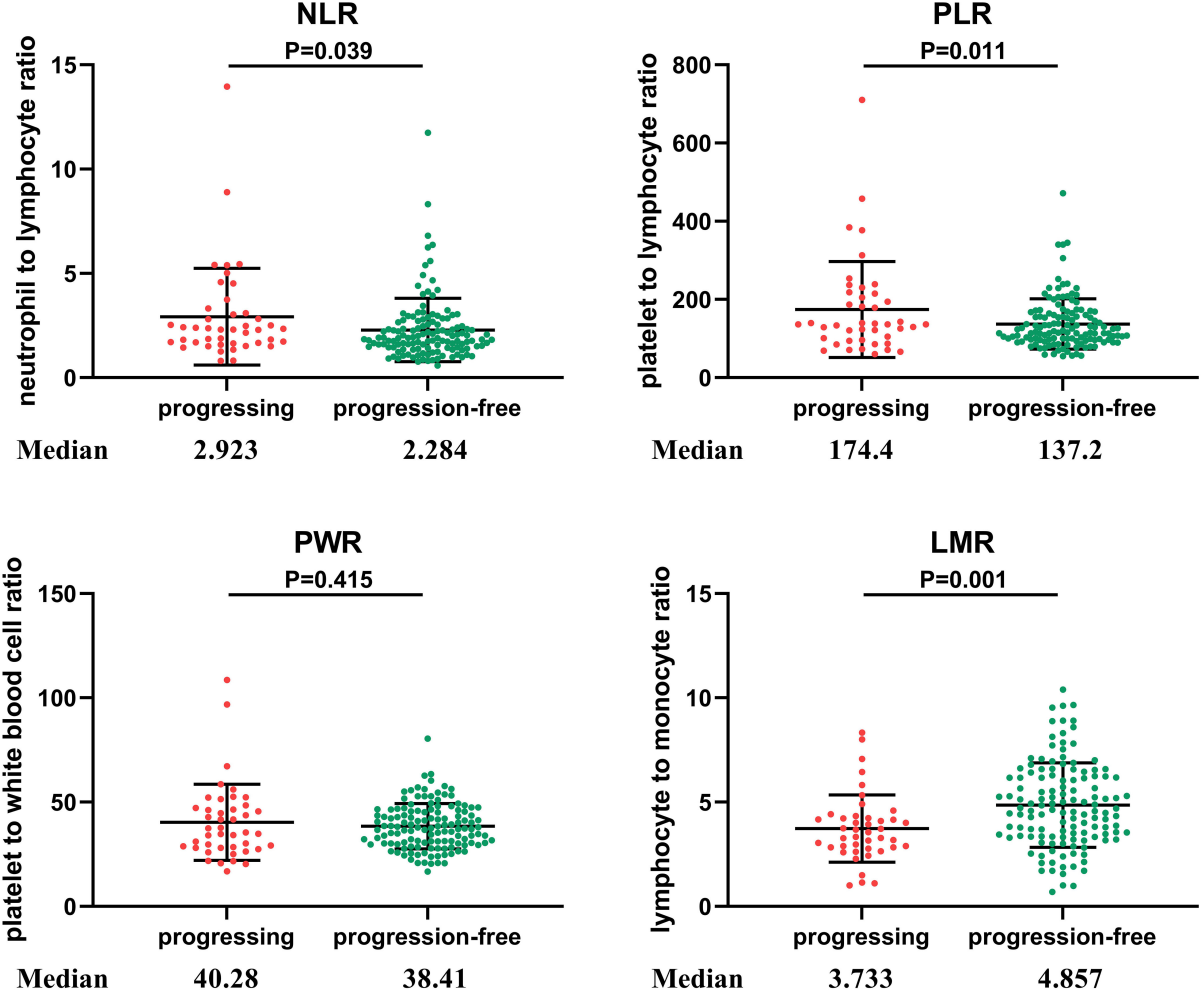
Distribution of inflammatory markers in PNETs. NLR, neutrophil to lymphocyte ratio; PLR, platelet to lymphocyte ratio; PWR, platelet to white blood cell ratio; LMR, lymphocyte to monocyte ratio. NLR was higher when tumor progression occurred relative to progression-free patients (*P* = 0.039), as was PLR (*P* = 0.011). LMR was lower in patients with progression than in those without progression (*P* = 0.001). No significant difference in PWR was found different between the two groups (*P* = 0.415).

**Figure 2:**
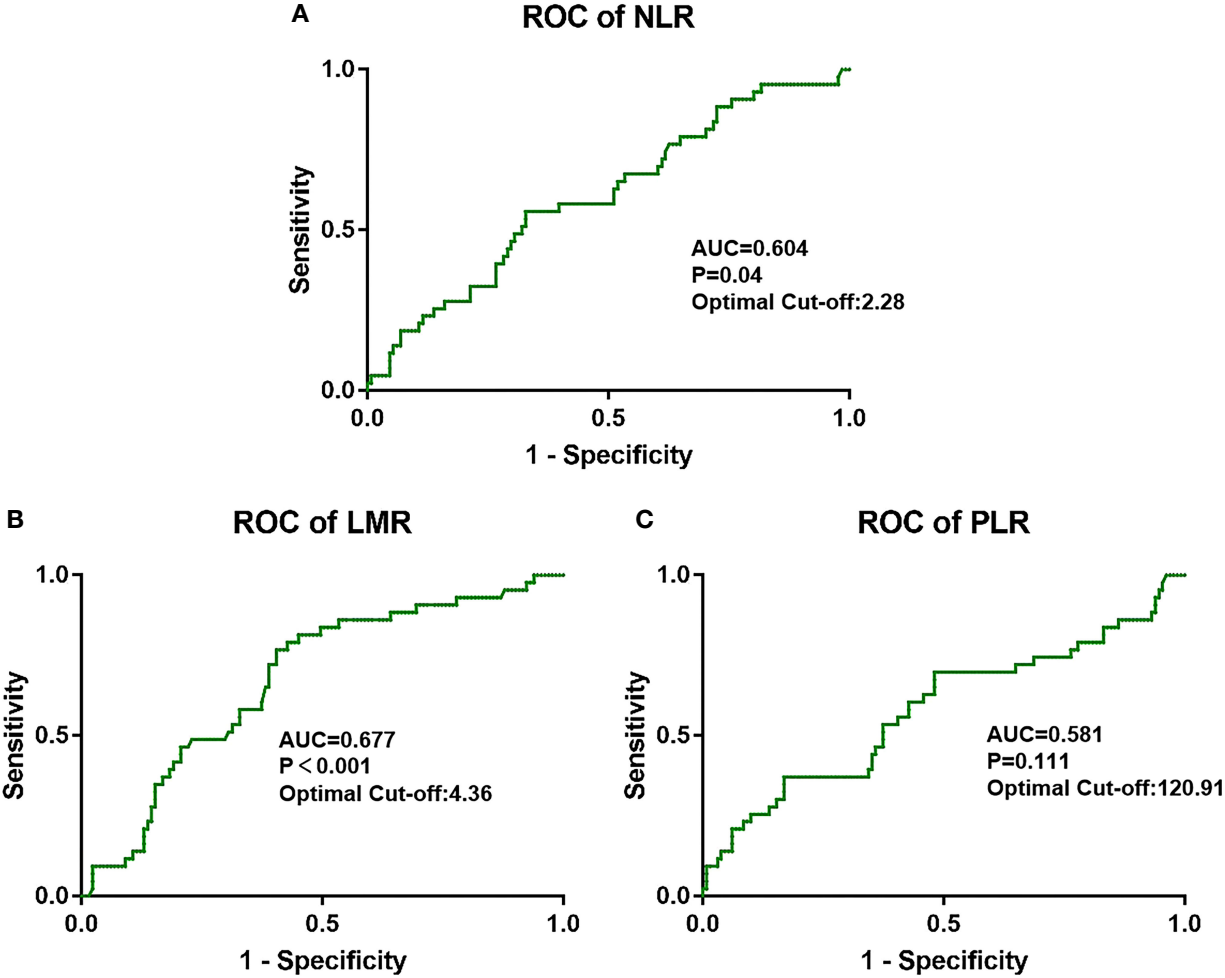
Optimal cutoff values for **(A)** NLR, **(B)** LMR, and **(C)** PLR and the defined area under the curve (AUC) were estimated from the receiver operating curve (ROC).

NLR was higher when tumor progression occurred relative to progression-free patients (median: 2.923 vs. 2.284, *P* = 0.039), as well as the PLR (median: 174.4 vs. 137.2, *P* = 0.011). LMR was lower in patients with progression than in those without (median: 3.733 vs. 4.857, *P* = 0.001). No significant difference in PWR was found (median: 40.28 vs. 38.41, *P* = 0.415; **Figure 1**). Cutoff values related to postoperative progression were determined from the ROC curve and gave optimal values of 2.28 for NLR, 4.36 for LMR, and 120.91 for PLR (**Figure 2**). Cutoff values enabled the patient cohort to be divided into high- and low-value groups. Higher proportions of tumors >2 cm (*P* = 0.001), more medium- to well-differentiated G2 and G3 tumors (*P* = 0.015), and significant correlation with TNM stage (*P* <0.001) were found among patients with high NLR. The low LMR group was associated with a greater number of smokers (*P* = 0.014) and worse tumor stage (*P* = 0.009). TNM stage was worse in the high PLR group (*P* = 0.016). No significant differences in gender, age, BMI, drinking history, tumor function, or tumor markers, such as CA125, CA199, and NSE, were present between the groups (**Table 2**).

**Table 2:** Comparison of the clinicopathological factors between the two groups classified by NLR, LMR, and PLR.

Clinicopathological factors	NLR			LMR			PLR		
	NLR ≥ 2.28 (*n* = 67)	NLR < 2.28 (*n* = 107)	*P*-value	LMR ≥ 4.36 (*n* = 84)	LMR < 4.36 (*n* = 90)	*P*-value	PLR ≥ 120.91 (*n* = 93)	PLR < 120.91 (*n* = 81)	*P*-value
Age (years)	51.76 ± 12.73	49.89 ± 13.03	0.353	49.67 ± 13.22	51.49 ± 12.62	0.354	50.89 ± 12.52	51.21 ± 13.39	0.568
Male/female	34/33	48/59	0.449	48/36	46/44	0.425	39/54	43/38	0.142
BMI (kg/m^2^)	23.57 ± 4.01	24.12 ± 3.43	0.361	23.74 ± 3.35	24.07 ± 3.94	0.568	23.92 ± 4.00	23.90 ± 3.25	0.968
Smoking, *n* (%)			0.400			**0.014**			0.294
Yes	9 (13.43%)	10 (9.35%)		4 (4.76%)	15 (16.67%)		8 (8.60%)	11 (13.58%)	
No	58 (86.57%)	97 (90.65%)		80 (95.24%)	75 (83.33%)		85 (91.40%)	70 (86.42%)	
Drinking, *n* (%)			0.625			0.847			0.725
Yes	5 (7.46%)	6 (12.36%)		5 (5.95%)	6 (6.67%)		7 (7.53%)	5 (6.17%)	
No	62 (96.74%)	101 (87.64%)		79 (94.05%)	84 (93.33%)		86 (92.47%)	76 (93.83%)	
Size (*n*, available)	61	104	**0.001**	81	84	0.471	87	78	0.064
>2 cm	38 (62.30%)	38 (36.54%)		35 (43.21%)	41 (48.81%)		46 (52.87%)	30 (38.46%)	
≤2 cm	23 (37.70%)	66 (63.46%)		46 (56.79%)	43 (51.19%)		41 (47.13%)	48 (61.54%)	
Subtype			0.524			0.418			0.833
Non-functioning	39 (58.21%)	57 (53.27%)		49 (58.33%)	47 (52.22%)		52 (55.91%)	44 (54.32%)	
Functioning	28 (41.79%)	50 (46.73%)		35 (41.67%)	43 (47.78%)		41 (44.09%)	37 (45.68%)	
Grade (*n*, available)	58	100	**0.015**	80	78	0.270	83	75	0.301
G1	25 (43.10%)	63 (63.00%)		48 (60.00%)	40 (51.28%)		43 (51.81%)	45 (60.00%)	
G2+G3	33 (45.90%)	37 (37.00%)		32 (40.00%)	38 (48.72%)		40 (48.19%)	30 (40.00%)	
Stage (*n*, available)	65	106	**<0.001**	83	88	**0.009**	91	80	**0.016**
I	14 (21.54%)	50 (48.11%)		34 (40.96%)	30 (34.09%)		27 (29.67%)	37 (46.25%)	
II	30 (46.15%)	40 (35.85%)		42 (50.60%)	28 (31.82%)		39 (42.86%)	31 (38.75%)	
III	3 (4.62%)	2 (2.83%)		1 (1.21%)	4 (4.54%)		5 (5.49%)	0 (0.00%)	
IV	18 (27.69%)	14 (13.21%)		6 (7.23%)	26 (29.55%)		20 (21.98%)	12 (15.00%)	
Progress, *n* (%)			**0.007**			**<0.001**			**0.013**
Yes	24 (35.82%)	19 (17.76%)		9 (10.71%)	34 (37.78%)		30 (32.26%)	13 (16.05%)	
No	43 (64.18%)	88 (82.24%)		75 (89.29%)	56 (62.22%)		63 (67.74)	68 (83.95%)	
Tumor markers									
CA125 (*n*, available)	53	91	0.055	70	74	0.398	77	67	0.183
Normal	46 (86.79%)	87 (95.60%)		66 (94.29%)	67 (90.54%)		69 (89.61%)	64 (95.52%)	
Abnormal	7 (13.21%)	4 (4.40%)		4 (5.71%)	7 (9.46%)		8 (10.39%)	3 (4.48%)	
CA199 (*n*, available)	53	91	0.204	70	74	0.141	77	67	0.482
Normal	47 (88.68%)	86 (94.51%)		67 (95.71%)	66 (89.19%)		70 (90.91%)	63 (94.03%)	
Abnormal	6 (11.32%)	5 (5.49%)		3 (4.29%)	8 (10.81%)		7 (9.09%)	4 (5.97%)	
NSE (*n*, available)	40	56	0.889	56	40	0.756	45	51	0.263
Normal	23 (57.50%)	33 (60.00%)		27 (90.91%)	29 (94.03%)		30 (95.71%)	26 (89.19%)	
Abnormal	17 (42.50%)	23 (40.00%)		18 (9.09%)	22 (5.97%)		26 (4.29%)	14 (10.81%)	

P-value <0.05 marked in bold font shows statistically significant differences.

PNETs, pancreatic neuroendocrine tumors; BMI, body mass index; NLR, neutrophil to lymphocyte ratio; LMR, lymphocyte to monocyte ratio; PLR, platelet to lymphocyte ratio; CA125, carbohydrate antigen 125; CA199, carbohydrate antigen 199; NSE, neuron-specific enolase.

### NLR, LMR, PLR, and prognosis

**Figure 3:**
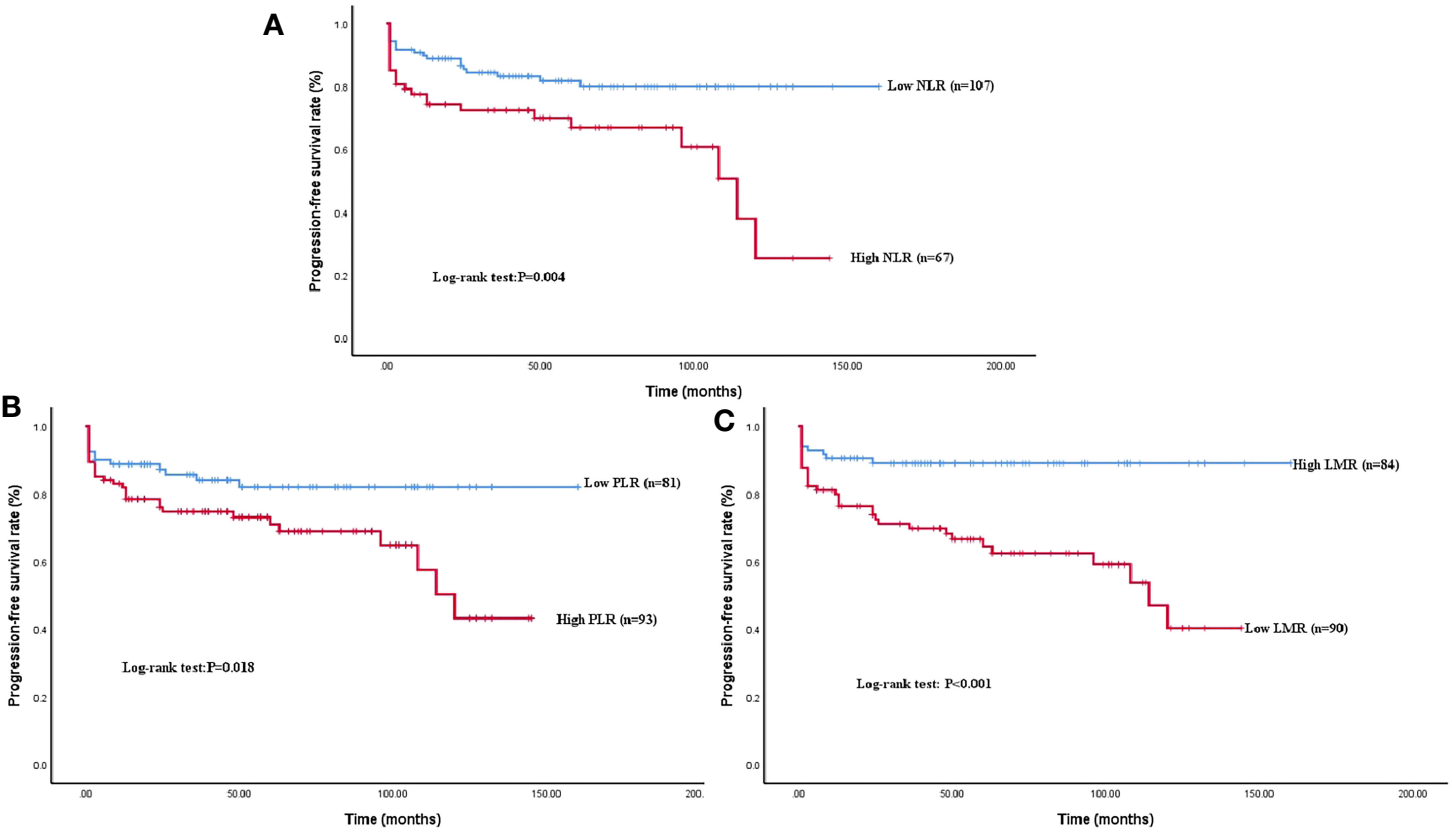
Kaplan–Meier survival curve analysis of PFS rate grouped by NLR, LMR, and PLR levels. A higher NLR **(A)**, a higher PLR **(B)**, and a low LMR **(C)** showed a significant correlation with shorter PFS (*P* = 0.004; *P* < 0.001; *P* = 0.018).

The median follow-up time was 47 months and the mean PFS of the whole cohort was 54 months. Forty-three patients (24.71%) experienced tumor progression after surgery (**Table 1**). The proportion of tumor progression in the high NLR (35.82% vs. 17.76%, *P* = 0.007), low LMR (37.78% vs. 10.71%, *P* <0.001), and high PLR (32.26% vs. 16.05%, *P* = 0.013) groups was higher than in the opposing groups (**Table 2**). Kaplan–Meier survival analysis demonstrated decreased survival among patients with high NLR (*P* = 0.004), low LMR (*P* < 0.001), and high PLR (*P* = 0.018) (**Figure 3**).

Single-factor Cox regression analysis showed that preoperative NLR, LMR, PLR, age, tumor function, tumor size, pathological grade, and CA199 were all significantly correlated with PFS. The risk of tumor progression was increased 2.352 times (95% CI: 1.286~4.303, *P* = 0.006) by high NLR, 3.742 times (95% CI: 1.794~7.804, *P* < 0.001) by low LMR, and 2.076 times by high PLR (95% CI: 1.083~3.982, *P* = 0.028). Statistically significant indicators were examined by multivariate Cox regression analysis. Preoperative LMR (HR: 3.128, 95% CI: 1.107~8.836, *P* = 0.031), pathological grade (HR: 5.433, 95% CI: 1.964~15.026, *P* = 0.001), and age (HR: 3.178, 95% CI: 1.214~8.320, *P* = 0.019) were all associated with PFS (**Table 3**). After adjustment for confounding factors, the postoperative risk of tumor progression was still greater in the low LMR than in the high LMR group.

**Table 3:** Univariate and multivariate analyses of PFS in patients with PNETs.

Variables	Univariate			Multivariate		
	HR	95% CI	*P*-value	HR	95% CI	*P*-value
Age (≥51 vs. <51)	2.961	1.509~5.810	**0.002**	3.178	1.214~8.320	**0.019**
Subtype (non-functioning vs. functioning)	0.397	0.203~0.776	**0.007**	1.288	0.465~3.568	0.626
Tumor size (>2 vs. ≤2)	2.815	1.399~5.664	**0.004**	1.465	0.541~3.965	0.453
Grade (G2, G3 vs. G1)	5.788	2.521~13.289	**<0.001**	5.433	1.964~15.026	**0.001**
NLR (high vs. low)	2.352	1.286~4.303	**0.006**	1.690	0.695~4.110	0.247
LMR (low vs. high)	3.742	1.794~7.804	**<0.001**	3.128	1.107~8.836	**0.031**
PLR (high vs. low)	2.076	1.083~3.982	**0.028**	0.610	0.228~1.632	0.325
CA125 (abnormal vs. normal)	0.826	0.198~3.453	0.794			
CA199 (abnormal vs. normal)	2.640	1.085~6.424	**0.032**	1.681	0.473~5.969	0.422

Only significant results (P < 0.05) from the univariate analysis are included in the multivariate analysis. P-value <0.05 marked in bold font shows statistically significant differences.

PFS, progression-free survival; PNETs, pancreatic neuroendocrine tumors; NLR, neutrophil to lymphocyte ratio; LMR, lymphocyte to monocyte ratio; PLR, platelet to lymphocyte ratio; CA125 carbohydrate antigen 125; CA199, carbohydrate antigen 199; HR, hazard ratio; CI, confidence interval.

## Discussion

PNETs are rare and account for less than 3% of all primary pancreatic tumors (1, 2). The rarity has limited the availability of data regarding clinical features and prognosis. A greater number of male PNET patients than female patients has been reported in the United States (19), but the current study suggests a slightly greater number of female patients with PNETs in Hubei Province, China. The proportion of the current cohort with comorbid diabetes was also not consistent with the conclusion of Zhuge et al. (20), suggesting an influence of race and region. Complications, including hypertension and other tumors, are also summarized by the current report and may give data useful for the treatment of this rare tumor. The heterogeneous nature of PNETs leads to great variation in individual prognoses, illustrating the importance of identifying prognostic indicators.

Inflammation is linked to tumor initiation, transformation, invasion, and metastasis (8, 21). Increased numbers of neutrophils, platelets, and monocytes and decreased lymphocytes are non-specific manifestations of tumor-related inflammatory reactions, associated with poor prognosis for a variety of malignant tumors (22–25). The inflammatory role of neutrophils promotes tumor growth, invasion, angiogenesis, and metastasis by secreting reactive nitrogen species (RNS), proteases, reactive oxygen species (ROS), vascular endothelial growth factor (VEGF), hepatocyte growth factor (HGF), and other cytokines (26–28). The systemic non-specific inflammatory response of thrombocytosis has long been recognized as an indicator of poor prognosis. Tumor cells interact with platelets to elicit hematogenous metastasis (29, 30). Macrophages derived from tumor-infiltrating monocytes secrete IL-10 and TGF-β to promote immunosuppression and mediate T lymphocyte dysfunction, allowing immune escape and tumor growth (31). The levels of peripheral blood lymphocytes reflect immune system activation, which acts to inhibit tumor cell proliferation and migration by activating cytotoxic lymphocytes (32, 33). Thus, diverse populations of blood cells regulate the balance between cancer, inflammation, and immunity. NLR and PLR are combined markers of inflammation and immune status, while LMR reflects antitumor immune activity. It can be seen that the level of NLR and PLR may be more susceptible to confounding factors. LMR may be more sensitive to the physiological state of the tumor.

The few studies on the markers of systemic inflammation in PNETs have focused on NLR, which has been shown to be an independent predictor of relapse-free survival (34). However, studies on the prognostic value of LMR have produced inconsistent findings. The current study analyzed the prognostic value of NLR, PLR, LMR, and PWR for PNET patients undergoing surgery. Single-factor Cox regression analysis showed high NLR and high PLR to be associated with tumor progression, reflecting the negative influence of a state of inflammation. However, NLR and PLR were not independently related to prognosis, suggesting the presence of confounding factors. Multivariate Cox analysis showed LMR to be an independent risk factor for predicting postoperative PNET progression. A cutoff value of LMR >4.36 had prognostic significance for identifying high-risk PNET patients.

We acknowledge some limitations to the current study. First, NLR, PLR, and LMR may be affected by inflammation, drugs, complications, and other factors. PNET patients are prone to surgical stress reactions and infection, which may affect the above indicators, but only preoperative levels were considered to minimize the interference of confounding factors. An in-depth exploration of complications and other confounding factors was outside the scope of the study. Secondly, this was a single-center, retrospective study, and the results may be biased. Finally, the sample size was small due to the low prevalence of PNETs. Therefore, a multicenter, large-sample prospective study is required for verification.

In conclusion, pathological tumor characteristics and predictors of postoperative PNET progression via accessible and low-cost blood tests were explored. The high risk of tumor progression and the merits of individualized treatment should be emphasized for patients with elevated preoperative inflammatory markers.

## Data availability statement

The original contributions presented in the study are included in the article/supplementary material, further inquiries can be directed to the corresponding author/s.

## Ethics statement

The studies involving humans were approved by the Committee for Medical Ethics of Union Hospital, Tongji Medical College, Huazhong University of Science and Technology. The studies were conducted in accordance with the local legislation and institutional requirements. Written informed consent for participation was not required from the participants or the participants’ legal guardians/next of kin.

## Author contributions

LYa: Writing – original draft. MF: Writing – original draft. LYu: Writing – original draft. HW: Writing – original draft. XC: Writing – original draft. HS: Writing – review & editing.
